# Characteristics of Multimodal Brain Connectomics in Patients With Schizophrenia and the Unaffected First-Degree Relatives

**DOI:** 10.3389/fcell.2021.631864

**Published:** 2021-02-25

**Authors:** Xiao Lin, WeiKai Li, Guangheng Dong, Qiandong Wang, Hongqiang Sun, Jie Shi, Yong Fan, Peng Li, Lin Lu

**Affiliations:** ^1^Peking University Sixth Hospital, Peking University Institute of Mental Health, Key Laboratory of Mental Health, Ministry of Health, National Clinical Research Center for Mental Disorders, Peking University, Beijing, China; ^2^College of Computer Science and Technology, Nanjing University of Aeronautics and Astronautics, Nanjing, China; ^3^Center for Cognition and Brain Disorders, Hangzhou Normal University, Hangzhou, China; ^4^Department of Psychology, Beijing Normal University, Beijing, China; ^5^National Institute on Drug Dependence and Beijing Key Laboratory on Drug Dependence Research, Peking University, Beijing, China; ^6^Department of Radiology, Perelman School of Medicine, University of Pennsylvania, Philadelphia, PA, United States; ^7^Peking-Tsinghua Center for Life Sciences and PKU-IDG/McGovern Institute for Brain Research, Peking University, Beijing, China

**Keywords:** schizophrenia, classification, magnetic resonance imaging, endophenotype, relatives

## Abstract

**Objective:**

Increasing pieces of evidence suggest that abnormal brain connectivity plays an important role in the pathophysiology of schizophrenia. As an essential strategy in psychiatric neuroscience, the research of brain connectivity-based neuroimaging biomarkers has gained increasing attention. Most of previous studies focused on a single modality of the brain connectomics. Multimodal evidence will not only depict the full profile of the brain abnormalities of patients but also contribute to our understanding of the neurobiological mechanisms of this disease.

**Methods:**

In the current study, 99 schizophrenia patients, 69 sex- and education-matched healthy controls, and 42 unaffected first-degree relatives of patients were recruited and scanned. The brain was parcellated into 246 regions and multimodal network analyses were used to construct brain connectivity networks for each participant.

**Results:**

Using the brain connectomics from three modalities as the features, the multi-kernel support vector machine method yielded high discrimination accuracies for schizophrenia patients (94.86%) and for the first-degree relatives (95.33%) from healthy controls. Using an independent sample (49 patients and 122 healthy controls), we tested the model and achieved a classification accuracy of 64.57%. The convergent pattern within the basal ganglia and thalamus–cortex circuit exhibited high discriminative power during classification. Furthermore, substantial overlaps of the brain connectivity abnormality between patients and the unaffected first-degree relatives were observed compared to healthy controls.

**Conclusion:**

The current findings demonstrate that decreased functional communications between the basal ganglia, thalamus, and the prefrontal cortex could serve as biomarkers and endophenotypes for schizophrenia.

## Introduction

Although numerous studies have been performed to discover the objective diagnostic biomarkers for schizophrenia, there is still remarkably less information available besides symptom-based assessments. The lifetime prevalence of schizophrenia is up to 1%, with a high contributing 13.4 million years of life lived with disability to the burden of disease globally (Charlson et al., 2018), which have been characterized by impaired semantics, hallucinations, delusions, loss of initiative, and impairments in cognitive functions (Owen et al., 2016). As a neuropsychiatric disorder, schizophrenia reflects the structural and functional changes of the brain; therefore, the well-developed neuroimaging approaches may provide potential biomarkers supporting an auxiliary diagnosis of this disease (Scognamiglio and Houenou, 2014; Voineskos, 2014). As a polygenic hereditary disorder, the approximately estimated heritability of schizophrenia is 0.8 (Sullivan et al., 2003), and it is hypothesized that the identification of the neuroimaging-based “endophenotypes,” which are more proximal to genetic influences than the illness itself (Winkler et al., 2010), will contribute to elucidating the biological mechanisms underlying schizophrenia and to identifying individuals at high risk.

The dysconnection hypothesis, which has been corroborated by structural and functional evidence, suggests that the dysconnectivity of functional brain networks, instead of a localized neural deficit, is linked to the neurological symptoms and cognitive impairments of schizophrenia (Vogeley and Falkai, 1998; Wagner et al., 2015; O’Neill et al., 2018). With the advance of neuroimaging techniques, studies have begun elucidating abnormalities in communications across brain regions and assessing the relationships between these abnormalities and the severity of the symptoms in schizophrenia (Fitzsimmons et al., 2013; Birur et al., 2017). Converging evidence from functional neuroimaging studies have revealed that functional connectivity of the prefrontal cortex (PFC) is reduced in schizophrenia (Zhou et al., 2015), with several independent studies reporting reduced intra-PFC connectivity in schizophrenia (Karbasforoushan and Woodward, 2012). A meta-analysis found hypoconnectivity in areas like the ventromedial PFC, hippocampus, and posterior cingulate cortex and, at the same time, hyperconnectivity/activity in the lingual gyrus in patients suffering from schizophrenia compared with healthy controls (Kühn and Gallinat, 2013). Even without obvious behavioral impairments, a decreased activity in the dorsal PFC was also observed in the unaffected relatives of patients with schizophrenia (Meda et al., 2008). Additionally, previous studies have frequently reported altered connectivity between the cortical and subcortical regions in schizophrenia, such as between the thalamus and cortex and between the posterior cingulate cortex and cerebellum (Byne et al., 2009). While dysconnectivity is strongly supported by empirical evidence from functional MRI, it is plausible to hypothesize that the connection problems originated in the disrupted structures. Enlarged lateral ventricles and reduced gray matter (GM) volume in the frontal operculum and lateral temporal lobes have been consistently reported (Wright et al., 2000; Horga et al., 2011). Recent advancement in diffusion MRI facilitates capturing subtle white matter abnormalities in schizophrenia, which cannot be detected by structural MRI (sMRI) alone. A recent meta-analysis revealed white matter bundle alterations consisting of callosal and commissural fibers, part of the motor descending fibers, and fronto-temporal-limbic pathways (Vitolo et al., 2017).

Most of the previous studies have focused only on single modalities, each of which has its own strengths and challenges (Jeurissen et al., 2011; Deloche et al., 2015). Different neuroimaging modalities provide different views of brain function or structure. Intuitively, the integration of multiple modalities may uncover the previously hidden information that cannot be found using any single modality. Given the limitations of single modalities, multimodal analysis provides a new avenue for reappraising the common beliefs of schizophrenia pathology and provides a full profile of the brain changes in mental disorders (Li et al., 2017, 2019, 2020). However, studies that combined these three modalities are still scarce. On the other hand, as a polygenic hereditary disorder, the approximately estimated heritability of schizophrenia is 0.8 and the SNP-based heritability is ∼20%, indicating that most of the heritability is unexplained. The brain alterations of the unaffected relatives of schizophrenia patients may deepen our understanding of this disease (Sullivan et al., 2003). It is hypothesized that shared neuroimaging abnormalities are considered genetically driven markers of risk. However, so far, the full profiles of the brain abnormalities of the at-risk yet healthy relatives of patients have not been fully exploited.

Very naturally, this study had three major objectives: (a) to determine whether the support vector machine (SVM) classifier established on integrated neuroimaging data from multimodalities can reliably distinguish patients and the unaffected first-degree relatives from healthy controls and to further verify the classifier in an independent sample; if so, (b) which regions show the highest discriminative power in discriminating schizophrenic patients and healthy subjects; and (c) to further investigate whether first-degree relatives share some overlapping abnormalities with schizophrenic patients.

## Materials and Methods

### Participants

Ninety-nine patients with chronic schizophrenia, 42 unaffected first-degree relatives of patients with schizophrenia, and 69 healthy control subjects were recruited for this study; the patients were identified at the Beijing Hui-Long-Guan Hospital, Beijing, China. The diagnosis of schizophrenia was made by one experienced psychiatrist (Zhao RJ) according to the Diagnosis and Statistic Manual of Mental Disorders, 4th edition (DSM-IV) criteria for schizophrenia using the Structured Clinical Interview for DSM-IV-TR Axis I Disorders, Patient Edition (SCID-I/P). All of the patients were evaluated for the severity of symptoms using the Positive and Negative Syndrome Scale (PANSS) within 1 week of the MRI scan. Of the 99 patients with schizophrenia, 19 were first-episode drug-naive patients, and 80 of them have been medicated (the drugs used include risperidone, olanzapine, aripiprazole, clozapine, quetiapine, ziprasidone, and amisulpride). To exclude the illness state-induced heterogeneity, an independent analysis was conducted on a sample that only included the 80 medicated patients (see Supplementary Table 5). The healthy controls did not have a family history of schizophrenia, personal psychiatric, or a neurological disorder. The following exclusion criteria were applied to all groups: (a) <18 or >45 years of age; (b) left-handedness; (c) history of brain trauma with loss of consciousness, neurological diseases, or serious physical diseases (such as respiratory disorders and cardiovascular disease); (d) diagnosis of alcohol/substance abuse within 12 months before participation; and (e) contraindications for MRI scan.

### Independent Sample

We used a separate dataset (from the website https://openfmri.org/dataset/ds000030/) to test whether the models of the classification are robust. This dataset included 171 participants, which consisted of 49 patients with schizophrenia (age, 36.13 ± 8.89 years; gender, 36 males) and 122 healthy controls (age, 31.59 ± 8.81 years; gender, 65 males).

### Neuroimaging Data Acquisition and Preprocessing

Resting-state functional MRI (fMRI) data, diffusion tensor imaging (DTI) data, and T1-weighted 3D high-resolution brain images were acquired for each subject on a Siemens MAGNETOM Trio 3.0-T imaging system with a standard head coil at the Peking University Third Hospital.

For image registration, high-resolution structural T1 MRI data were acquired with the following acquisition parameters: 256 × 256 matrix size, 192 contiguous axial slices, 1 mm slice thickness, 1 × 1 × 1 mm^3^ voxel resolution, 7° flip angle, 3.44 ms echo time, 2,530 ms repetition time, and 1,100 ms inversion time.

Resting-state functional scans were obtained using a gradient-recalled echo-planar imaging (GRE-EPI) sequence that was sensitive to blood oxygen level-dependent contrast (2,000 ms repetition time, 30 ms echo time, and 90° flip angle). The slice thickness was 4 mm (no gap) with a matrix size of 64 × 64 and a field of view of 220 × 220 mm^2^, resulting in a voxel size of 3.4 × 3.4 × 4.0 mm^3^. Each brain volume comprised 33 axial slices, and each functional run contained 240 image volumes. During data acquisition, the subjects were instructed to close their eyes, relax, and remain awake. The head movement represents a major confound, and there are two methods to detect excessively moving subjects in the present study. Firstly, the MRI machine we used has a monitor to tell the head movement, so patients with excessive motions were excluded during the data collection. Secondly, the preprocessing section also calculated the head motion and tried to exclude the patients with excessive head movements. Frame-wise displacement (FD) was calculated to measure volume-to-volume changes in head position. There was no significant difference among these groups in FD [*F*_(__2,210__)_ = 1.47, *p* = 0.23], and none of them had greater than 0.5 mm in FD scores.

Diffusion tensor imaging data were collected using a diffusion-weighted single-shot spin-echo planar imaging sequence with the following parameters: repetition time, 7,000 ms; echo time, 92 ms; field of view, 256 × 256 mm^2^; *b*_0_ image and 64 gradient directions at *b* = 1,000 s/mm^2^; matrix size, 128 × 128; voxel, size 2.0 × 2.0 × 3.0 mmł; and number of slices, 50.

The T1-weighted structural data were preprocessed using the VBM toolbox in SPM12 software^1^. The fMRI data were preprocessed by SPM12 and Data Processing and Analysis for Brain Imaging package^2^. The PANDA toolbox^3^ was adopted to preprocess the DTI data. Detailed descriptions of the data preprocessing procedures are in the Supplementary Material.

Quality control of the neuroimaging data was performed manually by visually inspecting the data at each step in the processing pipeline. To ensure a standard operation procedure, the fMRI scan will be performed with the same scanner and operator.

### Network Construction

The Human Brainnetome Atlas (Fan et al., 2016) was used to segment the brain into 246 regions (Supplementary Table 1 lists the labels, names, and abbreviations for these regions) to define the nodes of the brain networks. Specifically, the construction details of the morphological network, functional network, and DTI network are as follows: *Morphological networks* (T1): To quantify individual morphological relations of the brain regions, the Kullback–Leibler divergence-based similarity (KLS) measurement was utilized to construct networks based on the T1 data. The closer the GM density distribution of two brain regions, the higher is the KLS value [for a detailed description of this approach, please refer to Kong et al. (2014) and Wang et al. (2016)].

*Functional networks:* A time series of the low-frequency blood oxygenation level-dependent (BOLD) signals was extracted for each of the 246 regions and averaged over all voxels in each node. For each subject, the time series of all 246 regions were correlated with each other to create an undirected and weighted correlation matrix using Pearson’s correlation. These steps were performed with the CONN toolbox^4^. In contrast to partial correlation, the Pearson’s correlation coefficient is gaining higher values of reproducibility. In this network, each region represents a node with the correlation coefficients of the time series between the different regions defining the edges, resulting in a 246 × 246 connectivity matrix. No global signal regression was performed as it may result in a lower reproducibility of the network metrics.

*Anatomical networks* (DTI): The anatomical network of each subject was constructed by the DTI data using the probability tracking algorithm. Firstly, the T1-weighted structural images and the corresponding fractional anisotropy (FA) map were registered. Secondly, the Human Brainnetome Atlas template was registered into the individual DTI space. Thirdly, these 246 nodes were used as the seed points, respectively, to carry out probability tracking using the FDT of the FSL package. Typically, the connection probability of two brain regions has directivity (A→B is different from B→A). The connection probability *P*_*AB*_ is defined by the average of P_*A*__→__*B*_ and P_*B*__→__*A*_.

### Multiple-Kernel SVM Classifier

In this article, multiple kernel support vector machine (MK-SVM) was used, which can integrate multiple modalities of heterogeneous connection data (i.e., T1, fMRI, and DTI) for the individual classification of patients with schizophrenia (or first-degree relatives) from healthy controls (Zhu et al., 2014) since most of the classification studies of psychosis are focused on one modality and employed single-kernel SVM approaches. In general, each kernel is associated with a specific source of information and their combination is carried out to exploit complementary content coming from several features and modalities. Let us suppose there are *n* numbers of training samples and each of them is of the *M* numbers of modalities. Let $x_{i}^{m}$ represent a feature vector of the *m*-th modality of the *i*-th sample, and its corresponding class label be *yi* ∈ Error! Bookmarknot defined. Multiple-kernel-based SVM solves the following primal problem:

(1) $$\begin{array}{l} \min_{w^{m},b,\xi }\frac{1}{2}\sum_{m=1}^{M}\beta_{m}{||w^{m}||}^{2}+C\sum_{i=1}^{n}\xi_{i}\\ s.t.y_{i}\left(\sum_{m=1}^{M}\beta_{m}{\left(w^{m}\right)}^{T}\phi^{m}\left(x_{i}^{m}\right)b\right)\geq 1-\xi_{i}\\ \xi \geq_{i}=0,i=1,\cdots ,n \end{array}$$

where *w^m^*, ϕ^*m*^, and β_*m*_ ≥ 0 denote the normal vector of the hyperplane, the kernel-induced mapping function, and the combined weight on the *m*-th modality, respectively. As in the conventional SVM, the dual form of the multiple-kernel SVM can be represented as below:

(2) maxα∑i=1nαi-12∑i,jαiαjyiyj∑m=1Mβmkm(xim,xjm)s.t.∑i=1nαiyi=00≤α≤iC,i=1,2

where $k^{m}\left(x_{i}^{m},x_{j}^{m}\right)=\varphi^{m}{\left(x_{i}^{m}\right)}^{T}\varphi^{m}\left(x_{j}^{m}\right)$ is the kernel function for the two training samples on the *m*-th modality. The symbol *n* is the number of training samples. For a new test sample *x* = {*x*^1^, *x*^2^, …, *x^M^*}, $k^{m}\left(x_{i}^{m},x_{j}^{m}\right)=\varphi^{m}{\left(x_{i}^{m}\right)}^{T}\varphi^{m}\left(x^{m}\right)$ is firstly denoted as the kernel between the new test sample and each training sample on the *m*-th modality. Then, the decision function for the predicted label can be obtained as below:

(3) $$f\left(x_{1},x_{2},\ldots ,x_{M}\right)=\mathrm{sign}\left(\sum_{\text{i}=1}^{\text{n}}y_{i}\alpha_{i}\sum_{m=1}^{M}\beta_{m}k^{m}\left(x_{i}^{m},x^{m}\right)+b\right)$$

It is easy to show that the multiple-kernel-based SVM can be naturally embedded into the conventional single-kernel SVM if interpreted $k\left(x_{i},x_{j}\right)=\sum_{m=1}^{M}\beta_{m}k^{m}\left(x_{i}^{m},x_{j}^{m}\right)$ and *xj*, and $k\left(x_{i},x\right)=\sum_{m=1}^{M}\beta_{m}k^{m}\left(x_{i}^{m},x^{m}\right)$as a mixed kernel between the multimodal training sample *x*_*i*_ and the test sample *x*. In fact, our method can be viewed as a way for a kernel combination that combines multiple kernels into one kernel.

Different from the previous multi-kernel learning method, the present method constrains $\sum_{m=1}^{M}\beta_{m}=1$ and uses a coarse-grid search through cross-validation on the training samples to find the optimal values instead of jointly optimizing the weights β_*m*_ together with other SVM parameters in an iterative way. After obtaining the values of β_*m*_, these values were used to combine multiple kernels into a mixed kernel and then standard SVM was performed using the mixed kernel. This kernel combination method can provide a convenient and effective way of fusing various data from different modalities.

Subsequently, *t*-tests comparing every connection between the patients with healthy controls (and first-degree relatives with healthy controls) were performed separately for the training set, yielding a *p*-value for each connection. Since the dimensions might be different for the different modalities if we use the threshold methods, such as a *p*-value < 0.01, thus, we empirically selected the top 20 edges from each modality to avoid this impact. We used cross-validation to select the kernel weight of the different kernels or modalities, and this method is referenced to previous studies (Jie et al., 2014; Xu et al., 2020). For each modality, the top 20 significant connections were selected as input features for classification (*p* < 0.001), whereas the rest were eliminated. A leave-one-out cross-validation strategy was used, with inner cross-validation to determine the optimal parameters and outer cross-validation to determine the classification performance, as done in the previous study. The parameters, weight-combined mixed kernel, i.e., β_*m*_ in Eq. 1, of which classifier that showed the best classification performance were determined through the mentioned validation methods. A 10-fold cross-validation was also conducted to avoid overfitting. According to the different combinations, seven models were trained and summarized to compare the judgment effects of single modes and different modal combinations (including fMRI, DTI, sMRI, fMRI + DTI, fMRI + sMRI, DTI + sMRI, and fMRI + DTI + sMRI).

## Results

### Demographics

The demographic and clinical data are summarized in Table 1. The groups did not differ in gender or educational level. The first-degree relatives were older than the schizophrenia patients (SZs) and the healthy controls [*F*_(2_,_205)_ = 11.85, *p* < 0.05]. Significant differences were found across groups in the test scores for digit symbol coding (*p* < 0.05) and verbal fluency (*p* < 0.05). The SZs performed significantly worse on all of the cognitive tests compared with the healthy controls.

**TABLE 1:** Demographic and clinical features of the participants in each group.

Characteristic	Schizophrenia patients (*n* = 99)	First-degree relatives (*n* = 42)	Healthy control (*n* = 69)	Statistics	*p*
	Mean (SD)	Mean (SD)	Mean (SD)		
Age (years)	29.84 (8.55)	32.76 (7.97)	25.62 (5.95)	11.69	0.00
Sex (male/female)	39/60	17/25	38/31	4.43	0.11
Education (years)	12.68 (3.04)	11.6 (4.31)	13.35 (4.26)	2.33	0.10
Age of onset (years)	24.31 (7.52)				
Duration of illness (years)	5.58 (5.33)				
**PANSS score**					
Total	79.37 (7.33)				
Positive	26.12 (4.06)				
Negative	16.52 (3.69)				
General	35.73 (6.54)				
**Cognitive performance**					
Digit span	13.55 (3.30)	13.86 (9.41)	15.36 (3.36)	2.64	0.08
Verbal fluency	19.05 (6.51)	21.25 (5.75)	22.76 (5.18)	7.86	0.00
Digit symbol coding	44.61 (13.03)	57.63 (13.97)	64.06 (13.72)	43.73	0.00

### Networks and Classification

**FIGURE 1:**
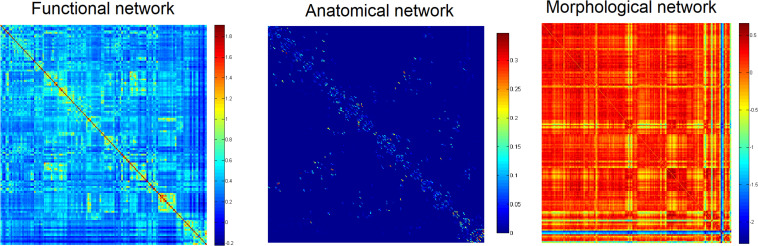
Group networks in healthy controls.

**FIGURE 2:**
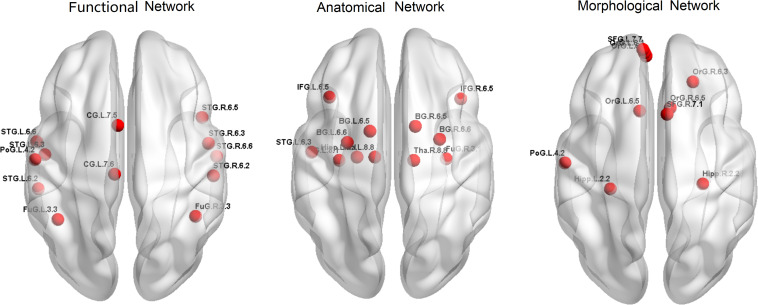
Hub regions for the three networks in healthy controls.

Firstly, to provide a profile of the multimodal networks, Figure 1 depicts the connectivity network for the healthy controls at the group level. The multimodal brain patterns we identified are consistent with a recent study, which also used multimodal analysis and found similar patterns in the healthy control group (Zhao et al., 2019). Figure 2 illustrates the hub regions in each network. The hub regions of the three major networks differed greatly. In the present study, two separate SVMs were utilized to classify patients from healthy controls and to classify unaffected relatives from healthy controls. For each classification, seven models were trained and summarized to compare the judgment effects of single modes and different modal combinations (including fMRI, DTI, sMRI, fMRI + DTI, fMRI + sMRI, DTI + sMRI, and fMRI + DTI + sMRI).

For the classification of patients and healthy controls, the functional network constructed using fMRI exhibited a relatively high accuracy rate (83.43%). The morphological networks constructed using the T1 image achieved an accuracy rate of 84.57%. The anatomical network constructed using the DTI data achieved the highest accuracy rate (89.71%). The classification accuracy was improved after combining the network features of the three modalities, achieving an accuracy of up to 94.86% with sensitivity and specificity of 92.86 and 96.19%, respectively. For the single-modality analyses, to classify relatives from healthy controls, the anatomical network constructed using the DTI data exhibited the highest accuracy rate (91.59%), followed by the functional network constructed using fMRI, which achieved an accuracy rate of 80.37%. The accuracy rate of the morphological network was 71.96%. Similarly, the classification accuracy was improved after combining the three modalities, achieving an accuracy of up to 95.33% with sensitivity and specificity of 94.29 and 91.90%, respectively; for details, see Table 2.

**TABLE 2:** Discrimination accuracy of the different models.

Data modality	SZ/HC			First-degree relatives/HC		
	Accuracy (%)	Sensitivity (%)	Specificity (%)	Accuracy (%)	Sensitivity (%)	Specificity (%)
fMRI	83.43	75.71	88.57	80.37	87.14	67.57
DTI	89.71	85.71	92.38	91.59	87.14	89.19
T1	84.57	80	87.62	71.96	84.29	48.65
fMRI + DTI	91.43	87.14	94.29	93.46	94.29	97.30
DTI + T1	90.29	84.29	94.29	83.18	90	70.27
fMRI + T1	92.57	90	94.29	90.65	90	91.89
fMRI + DTI + T1	94.86	92.88	96.19	95.33	94.29	91.90

**fMRI*, functional MRI; *DTI*, diffusion tensor imaging; *SZ*, patients with schizophrenia; *HC*, healthy controls.*

To test the generalizability of the models, validation (by direct application) of the prediction models should be tested in independent samples. We downloaded an open neuroimaging dataset from the OpenfMRI database (UCLA Consortium for Neuropsychiatric Phenomics^5^), which includes both healthy individuals (*n* = 122) and individuals with schizophrenia (*n* = 49). The classifier achieved an accuracy of up to 64.57% with sensitivity and specificity of 70 and 60.95%, respectively (for details, see Table 3). The multiple-kernel SVM classifier generated from the dataset of the present study was used to predict group membership of the cross-validation testing datasets. Each of the new datasets underwent identical preprocessing procedures to the original training cohorts. We predicted the group membership (SZ vs. healthy control) on these new subjects using the SVM models that were trained on the initial training cohort of subjects.

**TABLE 3:** Discrimination accuracy of the trained models in an independent sample.

Data modality	SZ/HC		
	Accuracy (%)	Sensitivity (%)	Specificity (%)
fMRI	51.43	94.29	25.71
DTI	62.28	52.86	68.57
T1	50.86	90	21.9
fMRI + DTI	52	90	26.67
DTI + T1	62.86	55.71	67.62
fMRI + T1	63.42	55.71	68.57
fMRI + DTI + T1	64.57	70	60.95

**fMRI*, functional MRI; *DTI*, diffusion tensor imaging; *SZ*, patients with schizophrenia; *HC*, healthy controls.*

### Discrimination Features

**FIGURE 3:**
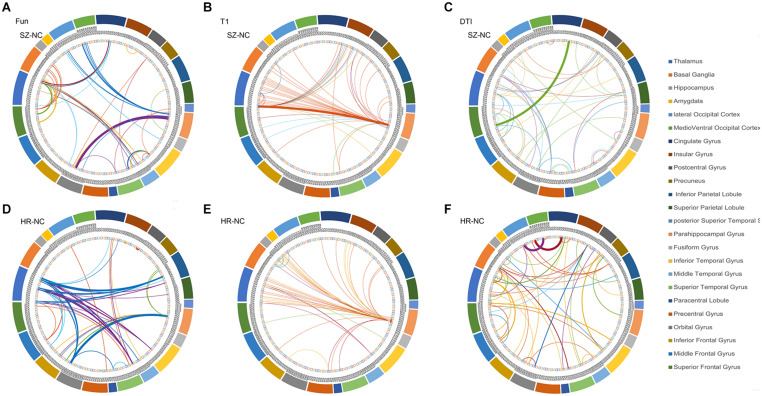
The features identified *via* the different modalities. **(A)** features used in functional network (SZ *vs.* HC), **(B)** features used in morphological network (SZ *vs.* HC), **(C)** features used in anatomical network (SZ *vs*. HC), **(D)** features used in functional network (first-degree relatives *vs.* HC), **(E)** features used in morphological network (first-degree relatives *vs.* HC), **(F)** features used in anatomical network (first-degree relatives *vs*. HC). *SZ*, patients with schizophrenia; *HC*, healthy controls.

The overlapped connections for the classification model that combined the three modal networks were examined. For each modality, the top 20 significant connections were selected as input features for classification (*p* < 0.001), whereas the rest were eliminated. However, in the cross-validation process, different top 20 significant connections were used for the different sample combinations. In Figure 3, all of the identified connections were displayed. To obtain a clearer point, 20 connections which showed the most significant contributions to the classifier with the best classification performance were selected for display (see Supplementary Tables 2–4). In the functional network, there were substantial overlapped changes between patients with schizophrenia and the unaffected first-degree relatives (see Figures 3A,B and Supplementary Table 2). There were 16 connections of the functional networks showing decreased functional connectivity in patients with schizophrenia compared with healthy controls, which were connections between the basal ganglia (BG) and superior frontal gyrus, para-hippocampus, and orbital frontal gyrus. Four of the decreased functional connections were also found in the unaffected first-degree relatives, whereas the functional connectivity between the thalamus and orbital frontal gyrus was increased in the patients and in the first-degree relatives. Frontal abnormal connections were revealed primarily with the insular, temporal, and BG regions.

All connections used in the feature set identified from the anatomical network (DTI) were decreased in the patient group compared with the healthy controls, and 12 of the connections showed decreased connectivity in the unaffected first-degree relatives (see Figures 3C,D and Supplementary Table 3). Most of the decreased connections were distributed within the PFC, with several connections between the BG and inferior frontal gyrus. The unaffected first-degree relatives showed an increase in connectivity between the inferior temporal gyrus and the superior frontal gyrus.

All of the connections identified from the morphological network (T1) found an increased connection for most of the connections in patients and in unaffected first-degree relatives compared to healthy controls (see Figures 3D,E and Supplementary Table 4). There were confidential overlaps between the increased connection in patients suffering from schizophrenia and in first-degree relatives, and most of the increased connections were located in the para-hippocampus, the thalamus, and the PFC.

## Discussion

This study investigated the intrinsic brain connectivity changes across multiple imaging modalities in patients with schizophrenia and their first-degree relatives. The results indicated that the convergent pattern was mostly located within the para-hippocampus and basal ganglia–thalamus–cortex circuit, and the features extracted from the three modalities yielded high classification accuracies both for the discrimination of patients and first-degree relatives.

### Decreased Functional Connections Between the Basal Ganglia, Para-Hippocampus, and Prefrontal Cortex in Patients and in First-Degree Relatives

In the present study, the functional network was constructed by connectivity strengths, which were estimated by Pearson’s correlation for each pair of brain regions, and yielded a diagnostic accuracy of approximately 83.43% for identifying the patients from healthy controls and a distinguishing accuracy of 80.37% for identifying the first-degree relatives. It was found that the schizophrenia group predominately exhibited weaker strengths of functional connectivity compared to the healthy group. In the exploration of the different brain regions, it was revealed that the serious dysconnectivities mainly appeared on the BG and PFC, especially the decreased functional connectivity within the PFC, which is consistent with previous studies (Zhou et al., 2015). There are plenty of studies that have reported the abnormal functional communication within the BG (Avram et al., 2018) and thalamus with cortex (Byne et al., 2009), although with less consistency. However, only a small number of studies have reported an altered BG function (including the midbrain) in schizophrenia. BG contains some of the most important dopamine regulatory regions, and an in-depth review (Chakravarthy et al., 2010) provided a comprehensive summary about the BG’s function [for more details, see Chakravarthy et al. (2010)]. Several studies have suggested that BG size can be a candidate biomarker to evaluate the effectiveness of antipsychotic response (Hutcheson et al., 2014). In patients with schizophrenia, the increase of BG volume is also related to D2 blockage due to antipsychotic administration (Zampieri et al., 2014). However, most of previous studies focused on the structural size of this area, and the brain connectivity has been scarcely investigated (Yoon et al., 2013). In the present study, the dysfunction of the communication between the PFC and BG is consistent with the hypothesis that BG inputs to the PFC act as a “gate” that typically mediates the behaviors. Although there are several divergent abnormalities in the relatives and patients, there are substantial overlapped alterations in functional connectives that could serve as endophenotypes. It was found that both the unaffected relatives and the patients exhibited weaker strengths of the functional connectivity between the para-hippocampus and PFC than healthy controls. A previous study revealed the decrease in volume of the para-hippocampus (Seidman et al., 2014) and PFC in the first-degree relatives of patients with schizophrenia (Faraone et al., 2003), and a decreased functional communication between the para-hippocampus and PFC was also reported during both rest and task (Eryilmaz et al., 2016). Contrary to the reduced functional connectivity between the thalamus and PFC in patients suffering from schizophrenia, the unaffected relatives showed an increase in functional connectivity between the thalamus and middle frontal gyrus.

### The Divergent Changes in Structural Connections Between Patients Suffering Schizophrenia and the First-Degree Relatives

The diagnostic accuracy of the anatomical network (DTI) reached as high as 89.71 and 91.59% for patients and for first-degree relatives, respectively. All of the connections used in the SVM were decreased in patients and most of them were decreased in first-degree relatives, most of which were the connections within the PFC and several were the connections between the subcortex and cortex. In a recent study, the investigators collected 4,322 samples from 29 datasets, reporting that FA values were generally lower among patients than among controls (Kelly et al., 2018). Surprisingly, the first-degree relatives showed several increased connections between the PFC with the thalamus and with the BG. The increased anatomical connections identified from the DTI were consistent with the increased functional connectivity between similar brain regions. The decreased functional and structural connections between the thalamus and PFC have been consistently reported in patients suffering from schizophrenia (Welsh et al., 2010; Marenco et al., 2012). The compensated increased functional communication between the thalamus and PFC may reflect protective brain features for the first-degree relatives from transition to illness. Contrary to a recently published study, the morphological networks resulted in the worst performance when discriminating the patients and the relatives from healthy controls. All of the connections used in this study were elevated, both in patients and in first-degree relatives, with the brain regions showing widespread distribution in the brain, consisting of the para-hippocampus, inferior prefrontal gyrus, and the thalamus. The covariant relationships between the largely distributed brain regions provide evidence for the largely affected brain regions.

So far, several studies have been conducted to investigate the brain connectivity changes using a multimodal neuroimaging method. A previous study combined the fractional amplitude of low-frequency fluctuations, GM, and FA measures, which suggested that the linked functional and structural deficits in the distributed cortico-striato-thalamic circuits may be closely related to cognitive impairments in schizophrenia (Sui et al., 2015). Xu et al. (2009) also identified four networks that were significantly associated with schizophrenia, including the temporal brain regions with the corpus callosum, frontal brain regions with the occipital fasciculus, the largely distributed frontal/parietal/occipital/temporal brain regions with the superior longitudinal fasciculus, and the parietal/frontal with the thalamus, reflecting the widespread nature of the disease. Despite the theoretical importance of investigating the brain abnormalities in unaffected first-degree relatives, few studies have investigated this issue by combining multimodal MRI features (Cooper et al., 2014). The present study focused on multimodal brain features in patients suffering from schizophrenia and in the unaffected first-degree relatives and found consistently decreased connections within the PFC and between the BG and cortex in relatives and patients compared to healthy controls. In comparison, the unaffected first-degree relatives showed an increase in structural and functional connectivity between the thalamus and PFC. This study suggested that the similarity between the unaffected relatives and patients suffering from schizophrenia reflects genetic influences on brain functions, which could serve as potential endophenotypes. In contrast, the opposite brain changes in relatives may serve as protective factors for the unaffected relatives.

### Limitations

The present study has some limitations. Firstly, most patients with schizophrenia enrolled in this study had taken antipsychotics; the effects of medication on the brain changes were not investigated here. Secondly, the age range is slightly different across three groups as the first-degree relatives and the enrolled patients were older than the healthy controls. However, considering the relatively small age range, we did not conduct further analysis to exclude the effect of age on out results.

## Conclusion

Although the connections identified by each modality differed, the convergent pattern is that all the brain connections identified and used to identify patients and relatives from healthy controls were mostly located within the para-hippocampus and the basal ganglia–thalamus–cortex circuit. The substantial overlaps between the patients and the unaffected first-degree relatives constitute candidate psychosis endophenotypes. The present study lends weight to previous suggestions that schizophrenia arises from the dysfunction of neural connectivity, which is sensitive to genetic influences.

## Data Availability Statement

The original contributions presented in the study are included in the article/Supplementary Material, further inquiries can be directed to the corresponding author/s.

## Ethics Statement

The studies involving human participants were reviewed and approved by the Beijing Hui-Long-Guan Hospital. The patients/participants provided their written informed consent to participate in this study.

## Author Contributions

PL and LL designed the experiment and wrote the first draft of the manuscript. PL and XL collected and analyzed the data. WL performed the multiple kernel SVM analysis and revised the first draft of the manuscript. HS, JS, and YF discussed the results and advised on interpretation. GD and QW contributed to the final draft of the manuscript. All authors contributed to and have approved the final manuscript.

## Conflict of Interest

The authors declare that the research was conducted in the absence of any commercial or financial relationships that could be construed as a potential conflict of interest.

## Footnotes

1 http://www.fl.ion.ucl.ac.uk/spm

2 http://www.rfmri.org/dpabi

3 https://www.nitrc.org/projects/panda/

4 https://web.conn-toolbox.org/

5 https://openfmri.org/dataset/ds000030/
