# Supplementary material for: Characteristics of Multimodal Brain Connectomics in Patients With Schizophrenia and the Unaffected First-Degree Relatives
Source: Front Cell Dev Biol. 2021 Feb 25;9:631864. doi: 10.3389/fcell.2021.631864 (PMC7947240; doi:10.3389/fcell.2021.631864)
Supplement: Supplementary file 1 [file Presentation_1.docx]

January 18, 2021, revised version for Front Cell Dev Biol (No. 631864)

**SUPPLEMENTARY MATERIAL**

**Characteristics of multimodal brain connectomics in patients with schizophrenia and the unaffected first-degree relatives**

Xiao Lin,^1, #^ WeiKai Li,^2, #^ Guangheng Dong,^3^ Qiandong Wang,^4^ Hongqiang Sun,^1^ Jie Shi,^5,^ Yong Fan,^6^ Peng Li,^1,^ * Lin Lu^1, 5, 7,^ *

^1^Peking University Sixth Hospital, Peking University Institute of Mental Health, Key Laboratory of Mental Health, Ministry of Health (Peking University), National Clinical Research Center for Mental Disorders (Peking University Sixth Hospital), Beijing 100191, China;

^2^College of Computer Science and Technology, Nanjing University of Aeronautics and Astronautics, Nanjing 211106, China;

^3^ Center for Cognition and Brain Disorders, Hangzhou Normal University, Hangzhou, Zhejiang Province, PR China;

^4^Department of Psychology, Beijing Normal University, Beijing, 100191, PR China;

^5^National Institute on Drug Dependence and Beijing Key Laboratory on Drug Dependence Research, Peking University, Beijing 100191, China;

^6^Department of Radiology, Perelman School of Medicine, University of Pennsylvania, Philadelphia, PA, 19104, USA.

^7^Peking-Tsinghua Center for Life Sciences and PKU-IDG/McGovern Institute for Brain Research, Peking University, Beijing 100871, China;

**^#^**These authors contributed equally

*These authors share senior authorship

Running Title: **Identifying brain connectomic biomarkers for schizophrenia**

Corresponding author:

Peng Li, M.D.

Peking University Institute of Mental Health

Key Laboratory of Mental Health

Peking University Sixth Hospital

38 Xue Yuan Road, Haidian District, Beijing 100191, China

E-mail: lipeng1986@bjmu.edu.cn

Tel: +86-10-62723738

Fax: +86-10-62032624

**Supplementary Methods and Materials**

1. T1 Data Preprocessing

The preprocessing was performed with the Diffeomorphic Anatomical Registration using Exponentiated Lie algebra (DARTEL) toolbox in Statistical Parametric Mapping (SPM12).The images were first segmented into gray matter, white matter and the cerebrospinal fluid. Then, these segmented images were spatially normalized and further modulated to preserve the absolute volume of gray matter. Finally, the data were smoothed spatially (FWHM 6 mm).

2. fMRI Data Preprocessing

The preprocessing was performed with SPM12 and Data Processing & Analysis for Brain Imaging package (DPABI: http://www.rfmri.org/dpabi). The first 10 volumes of the functional images were discarded for every individual participant to get stable fMRI signals. Then, slice-time corrected, motion-corrected, normalized, and temporal band-pass filtering (0.01–0.08 Hz) were conducted. Finally, the images were resampled to 2×2×2 mm³ during the normalization, and smoothed with a 6-mm full-width at half-maximum (FWHM) Gaussian kernel. After linear detrending, nuisance signal was regressed (including head motion parameters, the white matter, and cerebrospinal fluid signals).

3. DTI Data Preprocessing

The DTI data was pre-processed using the following steps: For each subject, DTI data were corrected for eddy currents and head motion by affine registration to the non-weighted image (b0 image); subsequently the skull from the data was removed; fractional anisotropy (FA) Fractional anisotropy was calculated and whole brain fractional anisotropy maps were normalized using a tissue probability map of white matter template, resampled to 2×2×2mm3 during normalization and spatially smoothed at 10mm FWHM. All these steps were performed using PANDA toolbox (PANDA: https://www.nitrc.org/projects/panda/).

| **Supplementary Table 1. Labels, names, and abbreviations for the 246 brain regions of Human Brainnetome Atlas** | | | | | | | |
| --- | --- | --- | --- | --- | --- | --- | --- |
| **Lobe** | **Gyrus** | **Left and Right Hemisphere** | **Label ID.L** | **Label ID.R** | **Anatomical and modified Cyto-architectonic descriptions** | **lh.MNI**  **(X,Y,Z)** | **rh.MNI**  **(X,Y,Z)** |
| **Frontal Lobe** | SFG, Superior Frontal Gyrus | SFG_L(R)_7_1 | 1 | 2 | *A8m, medial area 8* | -5 ,15, 54 | 7, 16, 54 |
|  |  | SFG_L(R)_7_2 | 3 | 4 | *A8dl, dorsolateral area 8* | -18, 24, 53 | 22, 26, 51 |
|  |  | SFG_L(R)_7_3 | 5 | 6 | *A9l, lateral area 9* | -11, 49, 40 | 13, 48, 40 |
|  |  | SFG_L(R)_7_4 | 7 | 8 | *A6dl, dorsolateral area 6* | -18, -1, 65 | 20, 4, 64 |
|  |  | SFG_L(R)_7_5 | 9 | 10 | *A6m, medial area 6* | -6, -5, 58 | 7, -4, 60 |
|  |  | SFG_L(R)_7_6 | 11 | 12 | *A9m,medial area 9* | -5, 36, 38 | 6, 38, 35 |
|  |  | SFG_L(R)_7_7 | 13 | 14 | *A10m, medial area 10* | -8, 56, 15 | 8, 58, 13 |
|  | MFG, Middle Frontal Gyrus | MFG_L(R)_7_1 | 15 | 16 | *A9/46d, dorsal area 9/46* | -27, 43, 31 | 30, 37, 36 |
|  |  | MFG_L(R)_7_2 | 17 | 18 | *IFJ, inferior frontal junction* | -42, 13, 36 | 42, 11, 39 |
|  |  | MFG_L(R)_7_3 | 19 | 20 | *A46, area 46* | -28, 56, 12 | 28, 55, 17 |
|  |  | MFG_L(R)_7_4 | 21 | 22 | *A9/46v, ventral area 9/46* | -41, 41, 16 | 42, 44, 14 |
|  |  | MFG_L(R)_7_5 | 23 | 24 | *A8vl, ventrolateral area 8* | -33, 23, 45 | 42, 27, 39 |
|  |  | MFG_L(R)_7_6 | 25 | 26 | *A6vl, ventrolateral area 6* | -32, 4, 55 | 34, 8, 54 |
|  |  | MFG_L(R)_7_7 | 27 | 28 | *A10l, lateral area10* | -26, 60, -6 | 25, 61, -4 |
|  | IFG, Inferior Frontal Gyrus | IFG_L(R)_6_1 | 29 | 30 | *A44d,dorsal area 44* | -46, 13, 24 | 45, 16, 25 |
|  |  | IFG_L(R)_6_2 | 31 | 32 | *IFS, inferior frontal sulcus* | -47, 32, 14 | 48, 35, 13 |
|  |  | IFG_L(R)_6_3 | 33 | 34 | *A45c, caudal area 45* | -53, 23, 11 | 54, 24, 12 |
|  |  | IFG_L(R)_6_4 | 35 | 36 | *A45r, rostral area 45* | -49, 36, -3 | 51, 36, -1 |
|  |  | IFG_L(R)_6_5 | 37 | 38 | *A44op, opercular area 44* | -39, 23, 4 | 42, 22, 3 |
|  |  | IFG_L(R)_6_6 | 39 | 40 | *A44v, ventral area 44* | -52, 13, 6 | 54, 14, 11 |
|  | OrG, Orbital Gyrus | OrG_L(R)_6_1 | 41 | 42 | *A14m, medial area 14* | -7, 54, -7 | 6, 47, -7 |
|  |  | OrG_L(R)_6_2 | 43 | 44 | *A12/47o, orbital area 12/47* | -36, 33, -16 | 40, 39, -14 |
|  |  | OrG_L(R)_6_3 | 45 | 46 | *A11l, lateral area 11* | -23, 38, -18 | 23, 36, -18 |
|  |  | OrG_L(R)_6_4 | 47 | 48 | *A11m, medial area 11* | -6, 52, -19 | 6, 57, -16 |
|  |  | OrG_L(R)_6_5 | 49 | 50 | *A13, area 13* | -10, 18, -19 | 9, 20, -19 |
|  |  | OrG_L(R)_6_6 | 51 | 52 | *A12/47l, lateral area 12/47* | -41, 32, -9 | 42, 31, -9 |
|  | PrG, Precentral Gyrus | PrG_L(R)_6_1 | 53 | 54 | *A4hf, area 4(head and face region)* | -49, -8, 39 | 55, -2, 33 |
|  |  | PrG_L(R)_6_2 | 55 | 56 | *A6cdl, caudal dorsolateral area 6* | -32, -9, 58 | 33, -7, 57 |
|  |  | PrG_L(R)_6_3 | 57 | 58 | *A4ul, area 4(upper limb region)* | -26, -25, 63 | 34, -19, 59 |
|  |  | PrG_L(R)_6_4 | 59 | 60 | *A4t, area 4(trunk region)* | -13, -20, 73 | 15, -22, 71 |
|  |  | PrG_L(R)_6_5 | 61 | 62 | *A4tl, area 4(tongue and larynx region)* | -52, 0, 8 | 54, 4, 9 |
|  |  | PrG_L(R)_6_6 | 63 | 64 | *A6cvl, caudal ventrolateral area 6* | -49, 5, 30 | 51, 7, 30 |
|  | PCL, Paracentral Lobule | PCL_L(R)_2_1 | 65 | 66 | *A1/2/3ll, area1/2/3 (lower limb region)* | -8, -38, 58 | 10, -34, 54 |
|  |  | PCL_L(R)_2_2 | 67 | 68 | *A4ll, area 4, (lower limb region)* | -4, -23, 61 | 5, -21, 61 |
| **Temporal Lobe** | STG, Superior Temporal Gyrus | STG_L(R)_6_1 | 69 | 70 | *A38m, medial area 38* | -32, 14, -34 | 31, 15, -34 |
|  |  | STG_L(R)_6_2 | 71 | 72 | *A41/42, area 41/42* | -54, -32, 12 | 54, -24, 11 |
|  |  | STG_L(R)_6_3 | 73 | 74 | *TE1.0 and TE1.2* | -50, -11, 1 | 51, -4, -1 |
|  |  | STG_L(R)_6_4 | 75 | 76 | *A22c, caudal area 22* | -62, -33, 7 | 66, -20, 6 |
|  |  | STG_L(R)_6_5 | 77 | 78 | *A38l, lateral area 38* | -45, 11, -20 | 47, 12, -20 |
|  |  | STG_L(R)_6_6 | 79 | 80 | *A22r, rostral area 22* | -55, -3, -10 | 56, -12, -5 |
|  | MTG, Middle Temporal Gyrus | MTG_L(R)_4_1 | 81 | 82 | *A21c, caudal area 21* | -65, -30, -12 | 65, -29, -13 |
|  |  | MTG_L(R)_4_2 | 83 | 84 | *A21r, rostral area 21* | -53, 2, -30 | 51, 6, -32 |
|  |  | MTG_L(R)_4_3 | 85 | 86 | *A37dl, dorsolateral area37* | -59, -58, 4 | 60, -53, 3 |
|  |  | MTG_L(R)_4_4 | 87 | 88 | *aSTS, anterior superior temporal sulcus* | -58, -20, -9 | 58, -16, -10 |
|  | ITG, Inferior Temporal Gyrus | ITG_L(R)_7_1 | 89 | 90 | *A20iv, intermediate ventral area 20* | -45, -26, -27 | 46, -14, -33 |
|  |  | ITG_L(R)_7_2 | 91 | 92 | *A37elv, extreme lateroventral area37* | -51, -57, -15 | 53, -52, -18 |
|  |  | ITG_L(R)_7_3 | 93 | 94 | *A20r, rostral area 20* | -43, -2, -41 | 40, 0, -43 |
|  |  | ITG_L(R)_7_4 | 95 | 96 | *A20il, intermediate lateral area 20* | -56, -16, -28 | 55, -11, -32 |
|  |  | ITG_L(R)_7_5 | 97 | 98 | *A37vl, ventrolateral area 37* | -55, -60, -6 | 54, -57, -8 |
|  |  | ITG_L(R)_7_6 | 99 | 100 | *A20cl, caudolateral of area 20* | -59, -42, -16 | 61, -40, -17 |
|  |  | ITG_L(R)_7_7 | 101 | 102 | *A20cv, caudoventral of area 20* | -55, -31, -27 | 54, -31, -26 |
|  | FuG, Fusiform Gyrus | FuG_L(R)_3_1 | 103 | 104 | *A20rv, rostroventral area 20* | -33, -16, -32 | 33, -15, -34 |
|  |  | FuG_L(R)_3_2 | 105 | 106 | *A37mv, medioventral area37* | -31, -64, -14 | 31, -62, -14 |
|  |  | FuG_L(R)_3_3 | 107 | 108 | *A37lv, lateroventral area37* | -42, -51, -17 | 43, -49, -19 |
|  | PhG, Parahippocampal Gyrus | PhG_L(R)_6_1 | 109 | 110 | *A35/36r, rostral area 35/36* | -27, -7, -34 | 28, -8, -33 |
|  |  | PhG_L(R)_6_2 | 111 | 112 | *A35/36c, caudal area 35/36* | -25, -25, -26 | 26, -23, -27 |
|  |  | PhG_L(R)_6_3 | 113 | 114 | *TL, area TL (lateral PPHC, posterior parahippocampal gyrus)* | -28, -32, -18 | 30, -30, -18 |
|  |  | PhG_L(R)_6_4 | 115 | 116 | *A28/34, area 28/34 (EC, entorhinal cortex)* | -19, -12, -30 | 19, -10, -30 |
|  |  | PhG_L(R)_6_5 | 117 | 118 | *TI, area TI(temporal agranular insular cortex)* | -23, 2, -32 | 22, 1, -36 |
|  |  | PhG_L(R)_6_6 | 119 | 120 | *TH, area TH (medial PPHC)* | -17, -39, -10 | 19, -36, -11 |
|  | pSTS, posterior Superior Temporal Sulcus | pSTS_L(R)_2_1 | 121 | 122 | *rpSTS, rostroposterior superior temporal sulcus* | -54, -40, 4 | 53, -37, 3 |
|  |  | pSTS_L(R)_2_2 | 123 | 124 | *cpSTS, caudoposterior superior temporal sulcus* | -52, -50, 11 | 57, -40, 12 |
| **Parietal Lobe** | SPL, Superior Parietal Lobule | SPL_L(R)_5_1 | 125 | 126 | *A7r, rostral area 7* | -16, -60, 63 | 19, -57, 65 |
|  |  | SPL_L(R)_5_2 | 127 | 128 | *A7c, caudal area 7* | -15, -71, 52 | 19, -69, 54 |
|  |  | SPL_L(R)_5_3 | 129 | 130 | *A5l, lateral area 5* | -33, -47, 50 | 35, -42, 54 |
|  |  | SPL_L(R)_5_4 | 131 | 132 | *A7pc, postcentral area 7* | -22, -47, 65 | 23, -43, 67 |
|  |  | SPL_L(R)_5_5 | 133 | 134 | *A7ip, intraparietal area 7(hIP3)* | -27, -59, 54 | 31, -54, 53 |
|  | IPL, Inferior Parietal Lobule | IPL_L(R)_6_1 | 135 | 136 | *A39c, caudal area 39(PGp)* | -34, -80, 29 | 45, -71, 20 |
|  |  | IPL_L(R)_6_2 | 137 | 138 | *A39rd, rostrodorsal area 39(Hip3)* | -38, -61, 46 | 39, -65, 44 |
|  |  | IPL_L(R)_6_3 | 139 | 140 | *A40rd, rostrodorsal area 40(PFt)* | -51, -33, 42 | 47, -35, 45 |
|  |  | IPL_L(R)_6_4 | 141 | 142 | *A40c, caudal area 40(PFm)* | -56, -49, 38 | 57, -44, 38 |
|  |  | IPL_L(R)_6_5 | 143 | 144 | *A39rv, rostroventral area 39(PGa)* | -47, -65, 26 | 53, -54, 25 |
|  |  | IPL_L(R)_6_6 | 145 | 146 | *A40rv, rostroventral area 40(PFop)* | -53, -31, 23 | 55, -26, 26 |
|  | Pcun, Precuneus | PCun_L(R)_4_1 | 147 | 148 | *A7m, medial area 7(PEp)* | -5, -63, 51 | 6, -65, 51 |
|  |  | PCun_L(R)_4_2 | 149 | 150 | *A5m, medial area 5(PEm)* | -8, -47, 57 | 7, -47, 58 |
|  |  | PCun_L(R)_4_3 | 151 | 152 | *dmPOS, dorsomedial parietooccipital sulcus(PEr)* | -12, -67, 25 | 16, -64, 25 |
|  |  | PCun_L(R)_4_4 | 153 | 154 | *A31, area 31 (Lc1)* | -6, -55, 34 | 6, -54, 35 |
|  | PoG, Postcentral Gyrus | PoG_L(R)_4_1 | 155 | 156 | *A1/2/3ulhf, area 1/2/3(upper limb, head and face region)* | -50, -16, 43 | 50, -14, 44 |
|  |  | PoG_L(R)_4_2 | 157 | 158 | *A1/2/3tonIa, area 1/2/3(tongue and larynx region)* | -56, -14, 16 | 56, -10, 15 |
|  |  | PoG_L(R)_4_3 | 159 | 160 | *A2, area 2* | -46, -30, 50 | 48, -24, 48 |
|  |  | PoG_L(R)_4_4 | 161 | 162 | *A1/2/3tru, area1/2/3(trunk region)* | -21, -35, 68 | 20, -33, 69 |
| **Insular Lobe** | INS, Insular Gyrus | INS_L(R)_6_1 | 163 | 164 | *G, hypergranular insula* | -36, -20, 10 | 37, -18, 8 |
|  |  | INS_L(R)_6_2 | 165 | 166 | *vIa, ventral agranular insula* | -32, 14, -13 | 33, 14, -13 |
|  |  | INS_L(R)_6_3 | 167 | 168 | *dIa, dorsal agranular insula* | -34, 18, 1 | 36, 18, 1 |
|  |  | INS_L(R)_6_4 | 169 | 170 | *vId/vIg, ventral dysgranular and granular insula* | -38, -4, -9 | 39, -2, -9 |
|  |  | INS_L(R)_6_5 | 171 | 172 | *dIg, dorsal granular insula* | -38, -8, 8 | 39, -7, 8 |
|  |  | INS_L(R)_6_6 | 173 | 174 | *dId, dorsal dysgranular insula* | -38, 5, 5 | 38, 5, 5 |
| **Limbic Lobe** | CG, Cingulate Gyrus | CG_L(R)_7_1 | 175 | 176 | *A23d, dorsal area 23* | -4, -39, 31 | 4, -37, 32 |
|  |  | CG_L(R)_7_2 | 177 | 178 | *A24rv, rostroventral area 24* | -3, 8, 25 | 5, 22, 12 |
|  |  | CG_L(R)_7_3 | 179 | 180 | *A32p, pregenual area 32* | -6, 34, 21 | 5, 28, 27 |
|  |  | CG_L(R)_7_4 | 181 | 182 | *A23v, ventral area 23* | -8, -47, 10 | 9, -44, 11 |
|  |  | CG_L(R)_7_5 | 183 | 184 | *A24cd, caudodorsal area 24* | -5, 7, 37 | 4, 6, 38 |
|  |  | CG_L(R)_7_6 | 185 | 186 | *A23c, caudal area 23* | -7, -23, 41 | 6, -20, 40 |
|  |  | CG_L(R)_7_7 | 187 | 188 | *A32sg, subgenual area 32* | -4, 39, -2 | 5, 41, 6 |
| **Occipital Lobe** | MVOcC*,* MedioVentral Occipital Cortex | MVOcC _L(R)_5_1 | 189 | 190 | *cLinG, caudal lingual gyrus* | -11, -82, -11 | 10, -85, -9 |
|  |  | MVOcC _L(R)_5_2 | 191 | 192 | *rCunG, rostral cuneus gyrus* | -5, -81, 10 | 7, -76, 11 |
|  |  | MVOcC _L(R)_5_3 | 193 | 194 | *cCunG, caudal cuneus gyrus* | -6, -94, 1 | 8, -90, 12 |
|  |  | MVOcC _L(R)_5_4 | 195 | 196 | *rLinG, rostral lingual gyrus* | -17, -60, -6 | 18, -60, -7 |
|  |  | MVOcC _L(R)_5_5 | 197 | 198 | *vmPOS,ventromedial parietooccipital sulcus* | -13, -68, 12 | 15, -63, 12 |
|  | LOcC, lateral Occipital Cortex | LOcC_L(R)_4_1 | 199 | 200 | *mOccG, middle occipital gyrus* | -31, -89, 11 | 34, -86, 11 |
|  |  | LOcC _L(R)_4_2 | 201 | 202 | *V5/MT+, area V5/MT+* | -46, -74, 3 | 48, -70, -1 |
|  |  | LOcC _L(R)_4_3 | 203 | 204 | *OPC, occipital polar cortex* | -18, -99, 2 | 22, -97, 4 |
|  |  | LOcC_L(R)_4_4 | 205 | 206 | *iOccG, inferior occipital gyrus* | -30, -88, -12 | 32, -85, -12 |
|  |  | LOcC _L(R)_2_1 | 207 | 208 | *msOccG, medial superior occipital gyrus* | -11, -88, 31 | 16, -85, 34 |
|  |  | LOcC _L(R)_2_2 | 209 | 210 | *lsOccG, lateral superior occipital gyrus* | -22, -77, 36 | 29, -75, 36 |
| **Subcortical Nuclei** | Amyg, Amygdala | Amyg_L(R)_2_1 | 211 | 212 | *mAmyg, medial amygdala* | -19, -2, -20 | 19, -2, -19 |
|  |  | Amyg_L(R)_2_2 | 213 | 214 | *lAmyg, lateral amygdala* | -27, -4, -20 | 28, -3, -20 |
|  | Hipp, Hippocampus | Hipp_L(R)_2_1 | 215 | 216 | *rHipp, rostral hippocampus* | -22, -14, -19 | 22, -12, -20 |
|  |  | Hipp_L(R)_2_2 | 217 | 218 | *cHipp, caudal hippocampus* | -28, -30, -10 | 29, -27, -10 |
|  | BG, Basal Ganglia | BG_L(R)_6_1 | 219 | 220 | *vCa, ventral caudate* | -12, 14, 0 | 15, 14, -2 |
|  |  | BG_L(R)_6_2 | 221 | 222 | *GP, globus pallidus* | -22, -2, 4 | 22, -2, 3 |
|  |  | BG_L(R)_6_3 | 223 | 224 | *NAC, nucleus accumbens* | -17, 3, -9 | 15, 8, -9 |
|  |  | BG_L(R)_6_4 | 225 | 226 | *vmPu, ventromedial putamen* | -23, 7, -4 | 22, 8, -1 |
|  |  | BG_L(R)_6_5 | 227 | 228 | *dCa, dorsal caudate* | -14, 2, 16 | 14, 5, 14 |
|  |  | BG_L(R)_6_6 | 229 | 230 | *dlPu, dorsolateral putamen* | -28, -5, 2 | 29, -3, 1 |
|  | Tha, Thalamus | Tha_L(R)_8_1 | 231 | 232 | *mPFtha, medial pre-frontal thalamus* | -7, -12, 5 | 7, -11, 6 |
|  |  | Tha_L(R)_8_2 | 233 | 234 | *mPMtha, pre-motor thalamus* | -18, -13, 3 | 12, -14, 1 |
|  |  | Tha_L(R)_8_3 | 235 | 236 | *Stha, sensory thalamus* | -18, -23, 4 | 18, -22, 3 |
|  |  | Tha_L(R)_8_4 | 237 | 238 | *rTtha, rostral temporal thalamus* | -7, -14, 7 | 3, -13, 5 |
|  |  | Tha_L(R)_8_5 | 239 | 240 | *PPtha, posterior parietal thalamus* | -16, -24, 6 | 15, -25, 6 |
|  |  | Tha_L(R)_8_6 | 241 | 242 | *Otha, occipital thalamus* | -15, -28, 4 | 13, -27, 8 |
|  |  | Tha_L(R)_8_7 | 243 | 244 | *cTtha, caudal temporal thalamus* | -12, -22, 13 | 10, -14, 14 |
|  |  | Tha_L(R)_8_8 | 245 | 246 | *lPFtha, lateral pre-frontal thalamus* | -11, -14, 2 | 13, -16, 7 |

**Supplementary Table 2. Feature connections used in machine learning within functional networks (fMRI).**

| Connection node 1 | Connection node 2 | First-degree relatives *minus* HC | Connection node 1 | Connection node 2 | SZ *minus* HC |
| --- | --- | --- | --- | --- | --- |
| 79 | 2 | 0.191204 | 219 | 2 | -0.16307 |
| 232 | 23 | 0.154703 | 220 | 2 | -0.18727 |
| 246 | 23 | 0.126566 | 223 | 2 | -0.2004 |
| 232 | 27 | 0.133854 | 224 | 2 | -0.19952 |
| 237 | 27 | 0.149238 | 226 | 2 | -0.18532 |
| 238 | 27 | 0.154672 | 230 | 2 | -0.18831 |
| 117 | 31 | -0.13756 | 231 | 5 | 0.176858 |
| 117 | 35 | -0.12886 | 232 | 5 | 0.189824 |
| 117 | 36 | -0.14202 | 229 | 10 | -0.20352 |
| 117 | 40 | -0.1571 | 230 | 10 | -0.22329 |
| 69 | 43 | -0.21424 | 231 | 41 | 0.175981 |
| 117 | 43 | -0.19683 | 232 | 41 | 0.177218 |
| 232 | 43 | 0.183848 | 157 | 44 | -0.1998 |
| 233 | 43 | 0.13274 | 117 | 45 | -0.21544 |
| 237 | 43 | 0.181193 | 118 | 45 | -0.20704 |
| 238 | 43 | 0.174389 | 70 | 46 | -0.20781 |
| 62 | 61 | -0.21257 | 117 | 46 | -0.19985 |
| 61 | 62 | -0.21257 | 118 | 46 | -0.20016 |
| 205 | 65 | 0.181891 | 118 | 50 | -0.18918 |
| 43 | 69 | -0.21424 | 46 | 70 | -0.20781 |

**Supplementary Table 3. Feature connections used in machine learning within anatomical networks (DTI).**

| Connection node 1 | Connection node 2 | First-degree relatives *minus* HC | Connection node 1 | Connection node 2 | SZ *minus* HC |
| --- | --- | --- | --- | --- | --- |
| 101 | 1 | 2.79E-06 | 20 | 5 | -0.00062 |
| 23 | 2 | -0.00071 | 241 | 5 | -3E-05 |
| 93 | 3 | 2.09E-06 | 11 | 6 | -0.00209 |
| 101 | 11 | 2.07E-07 | 239 | 6 | -3.2E-07 |
| 220 | 12 | 0.00014 | 6 | 11 | -0.00139 |
| 23 | 15 | -0.01415 | 12 | 11 | -0.01387 |
| 38 | 16 | 0.000438 | 14 | 11 | -0.00023 |
| 234 | 18 | 0.000521 | 180 | 12 | -0.04137 |
| 5 | 19 | -0.00644 | 50 | 13 | -1.7E-05 |
| 23 | 21 | -0.00592 | 239 | 16 | -3.6E-07 |
| 10 | 22 | -3.2E-05 | 164 | 18 | -1.8E-06 |
| 2 | 23 | -0.0027 | 5 | 20 | -0.00065 |
| 234 | 24 | 0.000295 | 11 | 20 | -0.00036 |
| 8 | 26 | -0.0227 | 239 | 20 | -9.7E-08 |
| 219 | 28 | 0.000212 | 23 | 21 | -0.00557 |
| 5 | 32 | -0.0001 | 7 | 23 | -0.00164 |
| 23 | 32 | -3.6E-05 | 74 | 30 | -4.3E-07 |
| 227 | 35 | -0.00194 | 131 | 34 | -1.9E-06 |
| 38 | 36 | 0.010148 | 5 | 35 | -0.00029 |
| 226 | 38 | -0.01386 | 2 | 36 | -2.5E-05 |

**Supplementary Table 4. Feature connections used in machine learning within morphological networks (T1).**

| Connection node 1 | Connection node 2 | First-degree relatives *minus* HC | Connection node 1 | Connection node 2 | SZ *minus* HC |
| --- | --- | --- | --- | --- | --- |
| 114 | 12 | 0.267422 | 114 | 246 | 0.316711 |
| 114 | 30 | 0.404323 | 114 | 245 | 0.295951 |
| 63 | 38 | 0.308542 | 114 | 218 | 0.352293 |
| 38 | 63 | 0.308542 | 72 | 114 | 0.337674 |
| 114 | 63 | 0.317969 | 114 | 235 | 0.30297 |
| 114 | 65 | 0.337598 | 114 | 236 | 0.26631 |
| 115 | 70 | 0.184912 | 114 | 232 | 0.206719 |
| 235 | 91 | 0.214353 | 114 | 244 | 0.14575 |
| 236 | 91 | 0.181015 | 114 | 240 | 0.153106 |
| 246 | 91 | 0.219487 | 103 | 246 | -0.15669 |
| 235 | 103 | 0.135681 | 114 | 243 | 0.14395 |
| 114 | 111 | 0.375587 | 173 | 246 | 0.316711 |
| 115 | 113 | 0.269784 | 114 | 223 | 0.295951 |
| 12 | 114 | 0.267422 | 238 | 246 | 0.352293 |
| 30 | 114 | 0.404323 | 172 | 246 | 0.337674 |
| 63 | 114 | 0.317969 | 114 | 239 | 0.30297 |
| 65 | 114 | 0.337598 | 114 | 221 | 0.26631 |
| 111 | 114 | 0.375587 | 238 | 244 | 0.232277 |
| 115 | 114 | 0.471933 | 114 | 169 | 0.374306 |
| 147 | 114 | 0.344329 | 175 | 246 | 0.310632 |

| Data modality | SZ/HC | | |
| --- | --- | --- | --- |
|  | Accuracy | Sensitivity | Specificity |
| fMRI | 85.81 | 78.57 | 92.31 |
| DTI | 74.32 | 74.29 | 74.36 |
| T1 | 75 | 77.14 | 73.08 |
| fMRI + DTI | 90.54 | 87.14 | 93.59 |
| DTI + T1 | 83.11 | 78.57 | 87.18 |
| fMRI + T1 | 86.49 | 82.86 | 89.74 |
| fMRI + DTI + T1 | 91.22 | 87.14 | 94.87 |

**Supplementary Table 5. Discrimination Accuracy of Different Models (%) (exclude the first-episode patients)**

**Supplementary Table 6. Discrimination Accuracy of Different Models (%) (10-fold cross-validation)**

| Data modality | SZ/HC | | |
| --- | --- | --- | --- |
|  | Accuracy | Sensitivity | Specificity |
| fMRI | 73.75 $\pm$0.69 | 70.71 $\pm$0.74 | 80.95 $\pm$0.61 |
| DTI | 84.39 $\pm$0.54 | 76.19 $\pm$0.58 | 87.88 $\pm$0.52 |
| T1 | 73.94 $\pm$0.77 | 75.12 $\pm$0.81 | 73.47 $\pm$0.72 |
| fMRI + DTI | 90.54 $\pm$0.65 | 86.15 $\pm$0.62 | 93.24 $\pm$0.69 |
| DTI + T1 | 82.11 $\pm$0.63 | 77.93 $\pm$0.61 | 87.18 $\pm$0.67 |
| fMRI + T1 | 85.64 $\pm$0.56 | 82.86 $\pm$0.51 | 90.35 $\pm$ 0.49 |
| fMRI + DTI + T1 | 92.19 $\pm$0.59 | 88.07 $\pm$0.53 | 93.24 $\pm$0.62 |

Mean $\pm$ SD are provided.

**Supplementary Table 7. Discrimination Accuracy of Different Models (%) (10-fold cross-validation)**

| Data modality | First-degree relatives /HC | | |
| --- | --- | --- | --- |
|  | Accuracy | Sensitivity | Specificity |
| fMRI | 75.67 $\pm$0.49 | 72.46 $\pm$0.56 | 80.95 $\pm$0.47 |
| DTI | 81.98 $\pm$0.60 | 79.71 $\pm$0.77 | 85.71 $\pm$0.56 |
| T1 | 73.87 $\pm$0.51 | 71.01 $\pm$0.56 | 78.57 $\pm$0.65 |
| fMRI + DTI | 89.18 $\pm$0.74 | 86.95 $\pm$0.98 | 92.85 $\pm$0.79 |
| DTI + T1 | 80.18 $\pm$0.42 | 76.81 $\pm$0.53 | 85.71 $\pm$0.51 |
| fMRI + T1 | 86.48 $\pm$0.81 | 85.50 $\pm$0.76 | 88.09 $\pm$ 0.93 |
| fMRI + DTI + T1 | 90.10 $\pm$0.69 | 88.40 $\pm$0.67 | 92.85 $\pm$0.64 |

Mean $\pm$ SD are provided.
